# Equivalence assessment of weight bearing cone beam CT and multidetector CT through 3D Knee bone modelling

**DOI:** 10.1038/s41598-025-06626-1

**Published:** 2025-07-01

**Authors:** Xiaoxu Li, Conrad Ivie, Philippe Van Overschelde, Sultana Monira Hussain, Khue Tran, Stuti Singh, Yu Peng

**Affiliations:** 1 Monash University, Clayton, 3800 Australia; 2 Tennessee Orthopaedic Alliance, Knoxville, 37923 United States; 3 Hip and Knee Clinic, Gent, 9830 Belgium; 4 Curvebeam AI, Melbourne, 3000 Australia; 5 University of Technology Sydney, Ultimo, 2007 Australia

**Keywords:** Weight-Bearing Computed Tomography, Multi-Detector Computed Tomography, 3D Knee Bone modelling, Patient CT, Cadaver-specimen CT, Biomedical engineering, Musculoskeletal system, Bone, Skeleton, Imaging techniques

## Abstract

Weight-bearing cone beam computed tomography (WB-CBCT, or simply WBCT), which captures high-resolution 3D images in a natural standing position, has gained increasing interest in recent years. This study examines the potential of WBCT as an alternative to multidetector computed tomography (MDCT) for 3D bone modelling. We generated 3D knee joint models from manually annotated WBCT and MDCT scans, performed rigid registration of these models, and assessed their similarity by evaluating the mean difference, standard deviation, and confidence intervals of the aligned models. The mean differences were computed as the average surface distances between corresponding WBCT and MDCT 3D bone models after rigid registration, providing a quantitative measure of their geometric similarity. Validation was conducted using both patient and cadaver scans to assess WBCT’s clinical applicability under realistic conditions and its technical reliability with controlled samples. Our findings reveal an average absolute difference of less than 0.35 mm for patient scans and 0.30 mm for cadaveric scans between WBCT and MDCT. The patella demonstrated the smallest mean difference (-0.20 mm to 0.10 mm) and standard deviation (0.28 mm to 0.55 mm) across all scans. These results confirm the comparability of WBCT to MDCT for 3D bone modelling, highlighting WBCT’s capacity to deliver appropriate image quality for the clinical assessment of bone joints.

## Introduction

Computed tomography (CT) has emerged as a gold standard in the clinical evaluation of bone structures. The derivation of 3D bone models from CT scans plays a pivotal role in orthopedic applications, facilitating the assessment of bone diseases and aiding in the planning of orthopedic surgeries. Among the various CT technologies, Multidetector CT (MDCT) has gained prominence due to its ability to provide detailed imaging at a variety of settings at any anatomical site. Recently, MDCT scanners capable of imaging in the upright position have been developed^1^, and several studies have been already published on knee evaluation using these upright MDCT scanners^2–6^. However, the availability of such devices remains limited. In parallel, the rapid advancement of weight-bearing cone beam CT (WB-CBCT, or simply WBCT) technology has sparked significant interest in recent years^7–9^. WBCT and captures high-resolution, 3D images while the patient is standing under full physiological load, providing critical insights into joint mechanics and musculoskeletal function. This weight-bearing position is critical for accurately assessing the musculoskeletal system under natural load conditions, uncovering potential issues that may not be visible in non-weight-bearing scans. WBCT’s relevance extends across orthopedics, podiatry, and sports medicine, offering insights that can shape treatment strategies, including surgical interventions and rehabilitation^10,11^.

WBCT employs cone-beam imaging technology, which utilizes a cone-shaped X-ray beam to capture data. This approach offers several advantages over the traditional fan beam of MDCT, including superior resolution, enhanced bone structure depiction, and reduced radiation exposure^12,13^. Nevertheless, cone-beam images typically exhibit lower contrast and greater noise than MDCT. To ascertain the clinical utility of WBCT-based 3D bone modelling, a comparison with MDCT-based models is imperative.

While several studies have compared 3D bone models across different imaging modalities, the focus has largely been on contrasting Magnetic Resonance (MR)-based models with those derived from MDCT, covering various body parts, including the knee^14,15^, teeth^16,17^, and skull^18^. For instance, Gao et al.^14^ measured the discrepancies between automatically segmented 3D MR bone models and manually segmented CT models of the pelvis, reporting an average surface distance of 1.24 mm. Neubert et al.^15^ assessed the 3D knee joint models from MR against CT-derived models, finding differences of less than 1 mm across all examined bones. However, studies specifically evaluating WBCT-based 3D bone models are scant, underscoring a need for validation against MDCT to reinforce the clinical applicability of WBCT.

This study aims to explore the viability of WBCT imaging as an alternative to MDCT for 3D bone modelling by comparing knee joint models derived from both WBCT and MDCT scans, encompassing both patient-scan and cadaver-specimen-scan assessments. The evaluation includes all four knee joint bones: the distal femur, proximal tibia, proximal fibula, and patella, comparing the 3D bone models generated through manual annotation of WBCT and MDCT scans. By measuring the 3D mesh similarity, this comparison allows for quantitative analysis and visualizing local discrepancies, facilitating a comprehensive analysis. To the best of our knowledge, this is the first study to validate the comparability of WBCT and MDCT via 3D bone models from knee joints.

## Materials and methods

### Image dataset

This study utilizes two distinct datasets to assess and compare the image quality derived from WBCT and MDCT. The first dataset comprises images from four individuals, while the second dataset consists of images from ten cadaver knees. This study is a retrospective analysis utilizing data originally acquired for robotic surgery planning. In clinical practice, robotic surgery systems commonly use slice thicknesses between 0.5 mm and 1 mm to balance spatial resolution and computational efficiency. The patient and cadaver scans were acquired by following the standard protocol used for robotic-assisted surgical planning at the time of acquisition. Since this is a retrospective study, the original acquisition settings—including slice thickness, field of view, radiation dose, and reconstruction algorithms—have been maintained without modification. Detailed descriptions of both datasets are provided below.

#### Ethics approval and consent to participate

All experimental protocols involving human participants were reviewed and approved by the Institutional Review Board of Advarra (Approval Reference: CR00386347, approved on 23 August 2022). All procedures were conducted in accordance with the ethical standards of the Institutional Review Board of Advarra and with the 1964 Helsinki Declaration and its later amendments or comparable ethical standards. Informed consent to participate in the study was obtained from all participants.

#### Patient dataset

The patient dataset was acquired using WB-CBCT and MDCT technologies (MDCT 1: GE LightSpeed VCT, United States; MDCT 2: Siemens SOMATOM Force CT, Germany). Four participants underwent knee joint scans at Parkwest Medical Center, Knoxville, United States. The time interval between the WBCT and MDCT scans for each participant was within ten days.

WBCT scans were captured with a kilovoltage of 120 kVp and an exposure of 43.2 mAs, resulting in a slice thickness of 0.60 mm and an in-plane resolution of 0.30 $\times$ 0.30 mm. The scan field was 400 mm (D, diameter) $\times$ 200 mm (H, height) and the Computed Tomography Dose Index volume (CTDIvol) was 1.95 mGy. For MDCT, the GE LightSpeed VCT scans (MDCT 1) were obtained with a kilovoltage of 120 kVp and an exposure of 403 mAs, featuring a slice thickness of 0.625 mm, an in-plane resolution of 0.49 $\times$ 0.49 mm. The scan field was 251 mm (D) $\times$ 376 mm (H) and the CTDIvol was 35.99 mGy. The Siemens SOMATOM Force CT scans (MDCT 2) were captured at 120 kVp, with an exposure ranging from 144 to 224 mAs, CTDIvol of 9.36 mGy and scan field of 250 mm (D) $\times$ 325 mm (H). The slice thickness was 1 mm, and the in-plane resolution was 0.48 $\times$ 0.48 mm. The acquisition parameters are summarized in Table 1.

**Table 1:** Acquisition parameters of the patient scans.

CT type	Kilovoltage (kVp)	Exposure (mAs)	Scan Field (mm)	CTDIvol (mGy)	Slice thickness (mm)	In-plane resolution (mm)
WBCT	120	43.2	400 D $\times$ 200 H	1.95	0.60	0.30 $\times$ 0.30
MDCT 1	120	403	251 D $\times$ 376 H	35.99	0.625	0.49 $\times$ 0.49
MDCT 2	120	144–224	250 D $\times$ 325 H	9.36	1	0.48 $\times$ 0.48

#### Cadaver specimen imaging dataset

The cadaver specimen dataset included scans of ten cadaver knees using both WB-CBCT and MDCT (Siemens SOMATOM go.Now CT, Germany). Each cadaver knee was scanned individually following a single-leg scanning protocol. Additionally, for WBCT, a bilateral-knee scanning setup was employed to mimic typical clinical scenarios. These scans were also performed at Parkwest Medical Center, Knoxville, United States.

WBCT images of the knees were acquired with a kilovoltage of 120 kVp and an exposure of 64.8 mAs, featuring a slice thickness of 0.50 mm and an in-plane resolution of 0.50 $\times$ 0.50 mm. The scan field was 440 mm (D) $\times$ 540 mm (H), and the CTDIvol was 2.75mGy. MDCT images were captured at 130 kVp with an exposure of 144 mAs, scan field of 440 mm (D) $\times$ 540 mm (H), and CTDIvol ranging from 1.51 to 2.75 mGy. The slice thickness was 1 mm and an in-plane resolution was 0.49 $\times$ 0.49 mm. The acquisition parameters for these scans are detailed in Table 2.

**Table 2:** Acquisition parameters of the cadaver specimen scans.

CT type	Kilovoltage (kVp)	Exposure (mAs)	Scan Field (mm)	CTDIvol (mGy)	Slice thickness (mm)	In-plane resolution (mm)
WBCT	120	64.8	440 D $\times$ 540 H	2.75	0.50	0.50 $\times$ 0.50
MDCT	130	144	250 D $\times$ 500 H	1.51${-}$ 2.64	1	0.49 $\times$ 0.49

### Bone segmentation

The distal femur, proximal tibia, proximal fibula, and patella were manually segmented from both WBCT and MDCT scans. This task was carried out by skilled experts using a combination of Stradview (University of Cambridge, Version 7.0) and 3D Slicer (Open Source, Version 4.11) software. To ensure accuracy and consistency, our manual segmentations underwent a multi-stage review process. Initially, three independent, experienced annotators segmented the bone masks. Each segmentation was then reviewed by two trained internal reviewers, who provided feedback and requested refinements as needed. The segmentation process continued iteratively until both reviewers reached a consensus on segmentation accuracy. Finally, an expert in medical image processing (PhD, >10 years of experience) conducted a final assessment and approved each segmentation before analysis. While segmentations were not explicitly repeated by the same annotator for variance measurement, this rigorous multi-review process ensured reliability across WBCT and MDCT segmentations.

**Fig. 1:**
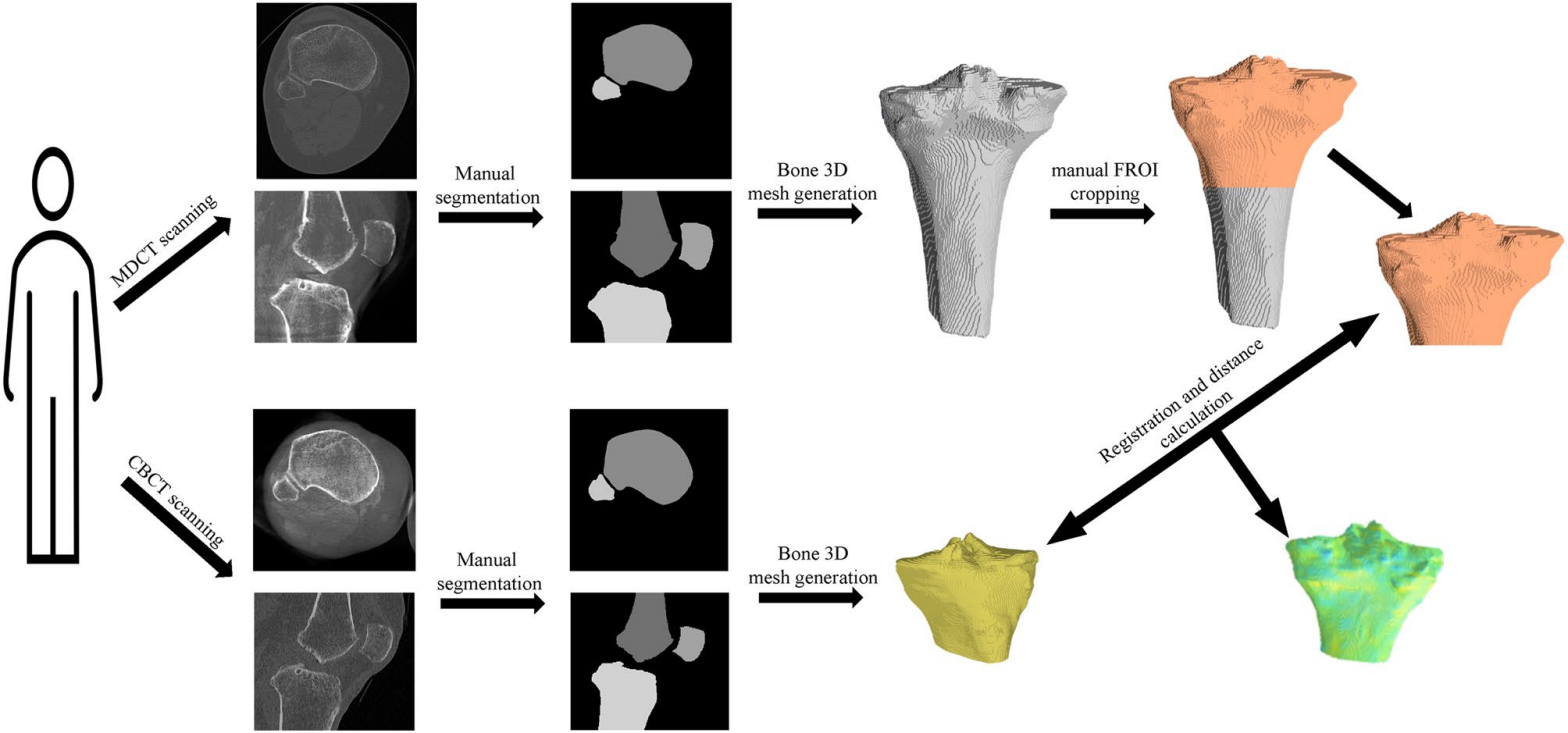
Overview of the pipeline illustrated with an example of the tibia (the process is identical for all bones). First, 3D meshes are extracted from manual segmentations of the individual bones in the WBCT and reference MDCT scans. The individual WBCT bone models are then rigidly registered to the corresponding MDCT bone model. A similarity metric for the bone surfaces is evaluated in an FROI defined in the reference MDCT bone mesh. The figure was created using Microsoft PowerPoint (version 16.77.1, https://www.microsoft.com/en-us/microsoft-365/powerpoint), ImageJ (version 1.52q, https://imagej.net/software/imagej/), Trimesh (version 3.21.7, https://trimesh.org/), and MeshLab (version 2022.02, https://www.meshlab.net/).

### 3D Bone modelling and comparison

As shown in Figure 1, the process of generating 3D bone models from WBCT and MDCT scans and their subsequent comparison involves several steps. Initially, the marching cubes algorithm^19^ is employed to transform image resolutions of WBCT and MDCT scans into real-world dimensions, factoring in pixel spacing and slice thickness. This algorithm facilitates the generation of 3D meshes from binary segmentation masks for each bone.

Subsequently, the iterative closest point (ICP) algorithm^20^ is applied for the rigid registration of each WBCT bone mesh to its MDCT counterpart to enable mesh distance calculations. This step is crucial, especially given the differences in field-of-views (FOVs) between WBCT and MDCT scans in patient scan imaging, necessitating the definition of focused regions-of-interest (FROIs) for each bone. FROIs are meticulously selected to ensure coverage of identical anatomical regions across WBCT and MDCT models for the distal femur, proximal tibia, and proximal fibula. The entire patella is included in both WBCT and MDCT scans, allowing direct comparison using the original bone meshes.

For the comparison, signed mesh distances between the registered WBCT and MDCT models are calculated within the FROIs. Distances are measured from the WBCT to the MDCT model, where each mesh vertex is assigned a positive or negative value to indicate whether the WBCT model is larger or smaller than the MDCT counterpart. These signed distances represent localized geometric differences between the two models at each vertex, while the mean surface distance across all vertices provides a global measure of geometric similarity. These metrics jointly enable the assessment of both regional shape discrepancies and overall alignment accuracy between the WBCT- and MDCT-derived 3D bone models. These differences are visualized using a standardized color map, ranging from red to blue, where red signifies the WBCT model is larger and blue indicates the MDCT model is larger.

In the patient dataset, a direct comparison is made between WBCT and MDCT-derived 3D models for each bone within the same participant. For the cadaver dataset, a similar pairing strategy is used: each cadaver knee has corresponding WBCT and MDCT scans, enabling direct comparison within the same specimen. Additionally, for cadavers with bilateral-leg WBCT scans, comparisons were also made with the single-leg MDCT scan of the same side to assess potential differences due to scan configuration.

## Results

### Results summary

The results from the patient and cadaver specimen scan with single-knee setup and bilateral-knee setup are summarized in Table 3, and the details are presented in Table 4, Table 5, and Table 6. As described in the Methods, these values do not correspond directly to the voxel size or CT slice thickness but rather result from post-processing the 3D surface models. After segmentation, bone surface models are exported as Standard Tessellation Language (STL) files, which represent the anatomical structures as interpolated triangular meshes. These STL meshes enable sub-voxel resolution for distance computation. The surface distance is calculated following rigid registration of the STL models, enabling for fine-scale measurement beyond the original CT voxel dimensions. The mean differences, standard deviations and confidence intervals across all datasets are reported in the tables.

**Table 3:** Results summary of patient and cadaver specimen scans (Unit: mm).

**Participant**	**Bones**	**Mean**	**Std**	**95CI** **upper** **bound**	**95CI** **lower** **bound**
Patient scans	Femur	0.006	0.07	0.15	−0.14
	Tibia	0.02	0.05	0.11	−0.07
	Fibula	−0.08	0.18	0.27	−0.43
	Patella	−0.04	0.03	0.03	−0.10
Cadaver scans single-knee setup	Femur	−0.03	0.11	0.18	−0.25
	Tibia	−0.09	0.10	0.11	−0.29
	Fibula	−0.13	0.07	−0.0004	−0.26
	Patella	−0.04	0.09	0.14	−0.22
Cadaver scans bilateral-knee setup	Femur	−0.05	0.17	0.29	−0.39
	Tibia	−0.13	0.11	0.08	−0.34
	Fibula	−0.12	0.07	0.02	−0.25
	Patella	−0.06	0.04	0.01	−0.13

**Patient Scans (Table **4) The patient data reveals minimal mean differences across participants and bones, highlighting a general equivalence in the imaging capabilities of WBCT and MDCT. The closeness of most mean values to zero underscores this comparability, especially for the patella, where the deviations are notably minimal. For instance, Participant 1’s femur shows a mere mean difference of 0.01 mm. The patella has an even smaller mean difference of 0.005 mm, underscoring the comparable image quality of both imaging modalities. The variability observed, particularly in the fibula measurements, suggests specific instances where image representation may diverge slightly between modalities. However, these variations are within a submillimeter range, reinforcing the notion that both WBCT and MDCT reliably capture bone structures in a clinical setting.

**Table 4:** Results of patient scans (Unit: mm).

**Participant**	**Bones**	**Mean**	**Std**	**95CI** **upper** **bound**	**95CI** **lower** **bound**
Participant 1	Femur	0.01	0.36	0.72	−0.69
	Tibia	0.07	0.38	0.81	−0.68
	Fibula	−0.35	0.83	1.28	−1.98
	Patella	0.005	0.33	0.65	−0.64
Participant 2	Femur	0.11	0.31	0.72	−0.49
	Tibia	0.05	0.50	1.03	−0.93
	Fibula	0.07	0.44	0.93	−0.79
	Patella	−0.07	0.45	0.81	−0.95
Participant 3	Femur	0.003	0.38	0.75	−0.74
	Tibia	0.003	0.38	0.75	−0.74
	Fibula	−0.12	0.54	0.94	−1.18
	Patella	−0.07	0.55	1.01	−1.15
Participant 4	Femur	−0.10	0.60	1.08	−1.28
	Tibia	−0.05	0.45	0.83	−0.93
	Fibula	0.09	0.26	0.60	−0.42
	Patella	−0.01	0.33	0.64	−0.66

**Cadaver specimen scan single-knee setup (Table **5) In the cadaver specimen scan single-knee setup, despite some variations in means and standard deviations, the overall data pattern suggests that both modalities effectively capture the details of bone anatomy. Notably, Cadaver 5’s patella reports a positive mean difference of 0.10 mm, indicating slight differences in image rendition that remain within the realm of comparability.

**Table 5:** Results of cadaver specimen dataset with single-knee setup (Unit: mm).

**Scans**	**Bones**	**Mean**	**Std**	**95CI** **upper** **bound**	**95CI** **lower** **bound**	**Scans**	**Bones**	**Mean**	**Std**	**95CI** **upper** **bound**	**95CI** **lower** **bound**
Cadaver 1	Femur	−0.05	0.44	0.81	−0.90	Cadaver 2	Femur	0.05	1.24	2.47	−2.38
	Tibia	−0.17	0.36	0.53	−0.88		Tibia	−0.23	0.44	0.63	−1.09
	Fibula	−0.16	0.40	0.63	−0.94		Fibula	−0.11	0.32	0.51	−0.73
	Patella	0.03	0.34	0.70	−0.64		Patella	−0.10	0.28	0.46	−0.66
Cadaver 3	Femur	0.05	0.43	0.90	−0.80	Cadaver 4	Femur	0.03	0.56	1.13	−1.07
	Tibia	0.04	0.37	0.77	−0.69		Tibia	−0.04	0.38	0.70	−0.78
	Fibula	−0.07	0.34	0.60	−0.74		Fibula	−0.11	0.30	0.49	−0.71
	Patella	0.01	0.31	0.62	−0.60		Patella	−0.09	0.31	0.51	−0.69
Cadaver 5	Femur	0.01	0.45	0.89	−0.86	Cadaver 6	Femur	−0.27	0.36	0.42	−0.97
	Tibia	−0.02	0.40	0.76	−0.80		Tibia	−0.16	0.30	0.44	−0.76
	Fibula	−0.06	0.34	0.61	−0.73		Fibula	−0.18	0.26	0.33	−0.70
	Patella	0.10	0.41	0.90	−0.70		Patella	−0.16	0.31	0.46	−0.78
Cadaver 7	Femur	−0.05	0.54	1.01	−1.10	Cadaver 8	Femur	0.11	1.09	2.24	−2.03
	Tibia	0.05	0.41	0.86	−0.75		Tibia	−0.06	0.41	0.74	−0.85
	Fibula	−0.16	0.44	0.71	−1.03		Fibula	−0.07	0.33	0.57	−0.71
	Patella	−0.02	0.40	0.76	−0.80		Patella	−0.02	0.38	0.72	−0.77
Cadaver 9	Femur	−0.18	0.38	0.55	−0.92	Cadaver 10	Femur	−0.04	0.37	0.68	−0.76
	Tibia	−0.26	0.39	0.50	−1.02		Tibia	−0.06	0.39	0.72	−0.83
	Fibula	−0.28	0.36	0.43	−0.99		Fibula	−0.08	0.41	0.72	−0.88
	Patella	−0.20	0.34	0.47	−0.87		Patella	0.06	0.32	0.69	−0.57

**Cadaver specimen scan bilateral-knee setup (Table **6) The results from the bilateral-knee setup further highlight the comparable imaging quality of WBCT and MDCT. The mean distance values reported for the tibia, fibula, patella, and femur across ten cadaver knees are notably minimal, from −0.30mm to 0.19mm. These minimal distances underscore the high fidelity of both WBCT and MDCT in capturing the intricate details of bone anatomy, even when both knees are imaged simultaneously, which is required in clinical assessments.

**Table 6:** Results of cadaver specimen dataset with bilateral-knee setup (Unit: mm).

**Scans**	**Bones**	**Mean**	**Std**	**95CI** **upper** **bound**	**95CI** **lower** **bound**	**Scans**	**Bones**	**Mean**	**Std**	**95CI** **upper** **bound**	**95CI** **lower** **bound**
Cadaver 1	Femur	0.15	0.99	2.08	−1.78	Cadaver 2	Femur	0.04	0.73	1.47	−1.38
	Tibia	−0.08	0.57	1.04	−1.20		Tibia	−0.17	0.43	0.67	−1.00
	Fibula	−0.06	0.50	0.92	−1.03		Fibula	−0.04	0.36	0.66	−0.74
	Patella	−0.09	0.39	0.67	−0.86		Patella	−0.06	0.32	0.58	−0.69
Cadaver 3	Femur	0.19	1.17	2.48	−2.10	Cadaver 4	Femur	0.11	1.05	2.18	−1.95
	Tibia	0.05	0.38	0.79	−0.70		Tibia	0.05	0.47	0.96	−0.86
	Fibula	−0.04	0.46	0.86	−0.94		Fibula	−0.02	0.46	0.88	−0.93
	Patella	−0.09	0.38	0.65	−0.83		Patella	−0.07	0.32	0.56	−0.69
Cadaver 5	Femur	−0.05	1.01	1.93	−2.02	Cadaver 6	Femur	−0.21	0.56	0.88	−1.30
	Tibia	−0.10	0.38	0.65	−0.85		Tibia	−0.08	0.37	0.65	−0.82
	Fibula	−0.15	0.28	0.41	−0.70		Fibula	−0.20	0.30	0.39	−0.79
	Patella	−0.04	0.47	0.89	−0.96		Patella	−0.12	0.39	0.64	−0.87
Cadaver 7	Femur	0.03	1.14	2.26	−2.21	Cadaver 8	Femur	−0.22	0.72	1.18	−1.62
	Tibia	−0.24	0.32	0.39	−0.87		Tibia	−0.27	0.48	0.68	−1.22
	Fibula	−0.19	0.30	0.40	−0.78		Fibula	−0.13	0.32	0.49	−0.76
	Patella	−0.05	0.28	0.50	−0.60		Patella	−0.04	0.32	0.58	−0.66
Cadaver 9	Femur	−0.30	0.89	1.44	−2.04	Cadaver 10	Femur	−0.25	0.79	1.29	−1.79
	Tibia	−0.23	0.41	0.58	−1.04		Tibia	−0.20	0.34	0.47	−0.87
	Fibula	−0.22	0.36	0.48	−0.92		Fibula	−0.14	0.32	0.49	−0.77
	Patella	−0.10	0.37	0.62	−0.83		Patella	0.01	0.28	0.56	−0.54

### Statistical equivalence analysis between MDCT and WBCT

A power analysis assuming a medium effect size (Cohen’s d = 0.5), an $\alpha$ level of 0.05, and a desired power of 0.80 indicated that a minimum of 34 samples would be required. In this study, all segmented bones (femur, tibia, fibula, and patella) were combined into a single dataset for statistical analysis, forming two comparison groups: patient plus single-leg cadaver and patient plus bilateral-leg cadaver. Each comparison group included 56 samples, exceeding the required sample size for detecting equivalence.

**Fig. 2:**
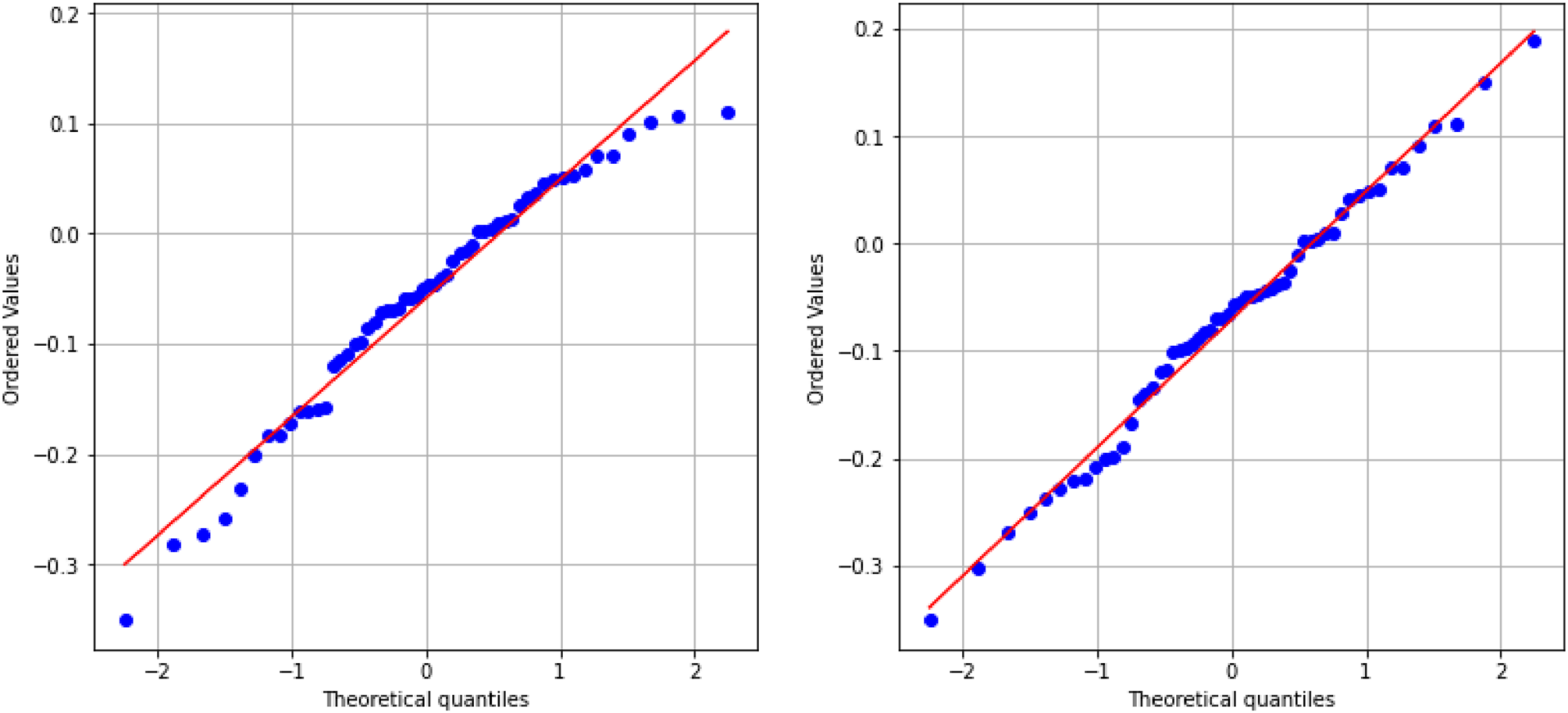
Q-Q plots comparing the observed mean surface distance data to a theoretical normal distribution. Left: Patient + Single Cadaver; Right: Patient + Bilateral Cadaver. The near-linear alignment of points along the 45-degree reference line indicated approximate normality of the data.

The normality of the mean surface distance data was assessed using both the Shapiro–Wilk and Kolmogorov–Smirnov tests, with results summarised in Table 7. For both comparison groups, no significant deviation from normality was detected (p > 0.05 for all tests). In addition, Quantile–Quantile (Q–Q) plots (Figure 2) demonstrated an approximately linear alignment along the reference line, supporting the assumption of normality.

**Table 7:** Normality testing results based on Shapiro-Wilk and Kolmogorov-Smirnov tests.

**Group**	**Shapiro-Wilk Test**			**Kolmogorov-Smirnov Test**		
	**Statistic (W)**	**P-value**	**Interpretation**	**Statistic (D)**	**P-value**	**Interpretation**
Patient + Single Cadaver	0.9639	0.0916	No significant deviation	0.0938	0.7021	No significant deviation
Patient + Bilateral Cadaver	0.9906	0.9417	No significant deviation	0.0758	0.9041	No significant deviation

**Table 8:** Results of TOST Equivalence Testing Between MDCT and WBCT.

**Group**	**Mean**	**Sample** ** Size**	**Equivalence** ** Margin (**±** mm)**	**TOST** ** Lower** ** P-Value**	**TOST** ** Upper** ** P-Value**	**95% CI** ** Lower**	**95% CI** ** Upper**	**Is** ** Equivalent**
Patient + Single Cadaver	−0.06	56	0.15	1.67E-08	6.71E-21	−0.08	−0.03	TRUE
Patient + Bilateral Cadaver	−0.07	56	0.15	2.37E-06	2.86E-20	−0.10	−0.04	TRUE

An equivalence test based on the Two One-Sided Tests (TOST) procedure was then performed, and an equivalence margin of ±0.15 mm was selected to represent the range of the smallest voxel size (0.3 mm) in this study. The results were summarized in Table 8. The analysis demonstrated that the mean differences across all comparison groups–including patient plus single-leg cadaver, and patient plus bilateral-leg cadaver–were statistically equivalent, with both lower and upper p-values < 0.05 and 95% confidence intervals well within the ±0.15 mm margin. These findings indicate that, from a clinical and statistical perspective, MDCT and WBCT produce equivalent 3D bone models for the knee joint.

### Results visualization

We provide a detailed visual comparison of 3D bone models derived from WBCT and MDCT scans in this section. The MDCT models of the femur, patella, tibia, and fibula serve as reference models. We employ a color-coding technique on the mesh vertices based on the signed mesh distance between each WBCT model and its MDCT counterpart. The visualization utilizes a color spectrum ranging from dark blue to dark red, representing signed mesh distances from negative to positive. A positive distance indicates that the WBCT model protruded beyond the MDCT model, suggesting a “larger” appearance, whereas a negative value indicates the opposite. The intensity of the color increases with the absolute value of the distance, with red highlighting areas where the WBCT model is larger and blue for where the MDCT model is larger.

The signed mesh distance visualization was employed to provide a spatially detailed comparison between WBCT and MDCT-derived 3D bone models. This method allows for the identification of localized discrepancies in surface modelling, which cannot be easily captured through summary statistics alone. By applying a standardized color map, the distribution of differences across the entire bone structure could be visually assessed, enabling a more intuitive interpretation of the agreement between the two imaging modalities. This approach has been widely used in comparative imaging studies to assess morphological deviations and ensure clinical applicability^15,21^.

**Fig. 3:**
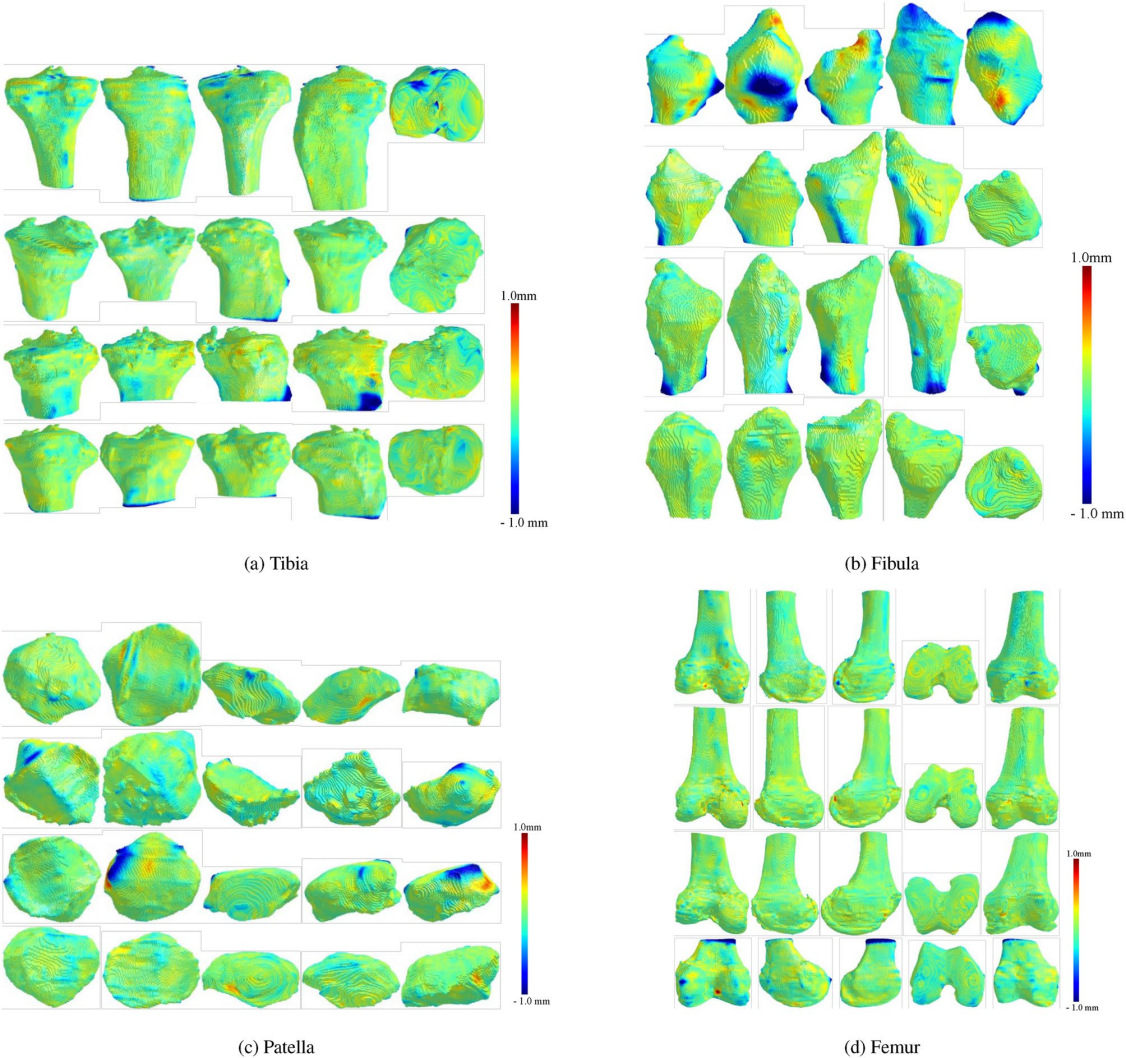
Results of the patient dataset for (**a**) Tibia, (**b**) Fibula, (**c**) Patella, and (**d**) Femur. Visual comparison of the differences between WBCT and MDCT bone models using the signed difference metric. The metric is presented using the MDCT model as a reference surface to allow direct visual comparisons between the WBCT models. A positive metric sign (toward red colors) means that the WBCT model extends beyond the MDCT model, whereas a negative metric sign (toward blue colors) means that the MDCT model extends beyond the WBCT. The figure was created using Microsoft PowerPoint (version 16.77.1, https://www.microsoft.com/en-us/microsoft-365/powerpoint), Trimesh (version 3.21.7, https://trimesh.org/), and Matplotlib (version 3.7.1, https://matplotlib.org/).

**Fig. 4:**
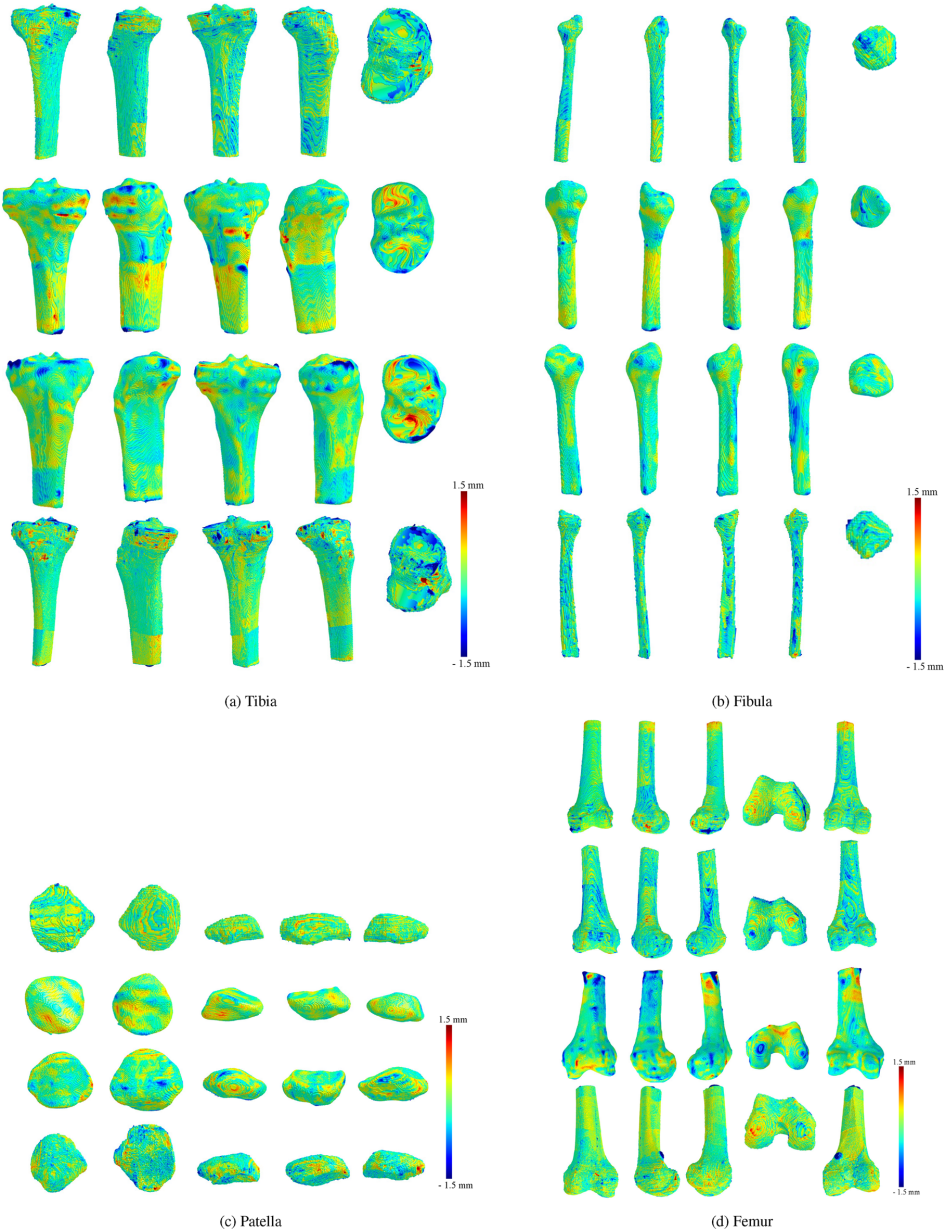
Results of cadaver specimen dataset for (**a**) Tibia, (**b**) Fibula, (**c**) Patella, and (**d**) Femur. The first two rows in each image show single-knee WBCT scanning, and the last two rows show bilateral-knee WBCT scanning. The figure was created using Microsoft PowerPoint (version 16.77.1, https://www.microsoft.com/en-us/microsoft-365/powerpoint), Trimesh (version 3.21.7, https://trimesh.org/), and Matplotlib (version 3.7.1, https://matplotlib.org/).

To assess the registration differences between the WBCT and MDCT 3D models, we prepare five distinct views for each bone model in the patient and cadaver specimen datasets. These views are illustrated in Figure 3 for the patient dataset and Figure 4 for the cadaver specimen dataset. A significant observation from these visual comparisons is the predominance of very light colors across the surface of each bone model, denoting minimal discrepancies between the WBCT models and their MDCT counterparts. A more in-depth discussion and analysis of these visual findings are presented in the following two sections, where we delve into the implications of these minimal differences.

### Results of patient scans

#### Tibia results

Analysis of Table 4 reveals that the average absolute distance between the WBCT and MDCT models of the tibia is consistently small (< 0.1mm) for all participants, with standard deviations ranging from 0.38mm to 0.50mm. Though the darker hues near the top of the neck area and some convex plateaus in Figure 3a suggest localized differences, the visualization demonstrates a predominance of light coloring across the tibia surface, indicating minor discrepancies.

#### Fibula results

The fibula shows an average absolute distance of around 0.1mm, excluding the first participant who presents a slightly higher deviation (0.35mm), with standard deviations between 0.26mm and 0.54mm (Table 4). As illustrated in Figure 3b, the surfaces of the fibula in three participants are predominantly light-colored, indicating minimal variation. The first participant’s fibula, despite its greater average distance, exhibits most discrepancies along the lateral convex borders, with the majority of the surface displaying light coloring.

#### Patella results

Given that the patella bones are fully included in our scans, no FROI extraction is necessary, eliminating potential registration errors from this process. All participants show average absolute distances below 0.1mm, with the smallest deviations (0.01mm) observed in the first and fourth participants. The standard deviation ranges from 0.33mm to 0.55mm, with the highest variability seen in the third participant. Visualization (Figure 3c) highlights a concentrated area of variance along the lateral border of the third participant’s patella, contrasting with the less pronounced discrepancies in other participants.

#### Femur results

The femur exhibits generally small average absolute distances ($\sim$0.1mm for the second and fourth participants; 0.01mm for the first and third), with standard deviations from 0.31mm to 0.60mm (Table 4). Visualization results (Figure 3d) reveal isolated areas of darker coloring for the first three participants, indicating minor differences. The fourth participant’s femur, however, displays more significant dark regions along the FROI borders, suggesting areas of greater discrepancy due to the FROI extraction process.

### Results of cadaver specimen scans

#### Results of single-knee WBCT scanning

Table 5 outlines the cadaver specimen single-knee WBCT scan results, showcasing similarities to the patient findings. The average distances for the tibia, fibula, patella, and femur are notably consistent, with all absolute values below 0.28mm, and standard deviations indicating a normal distribution of differences across bones. Visualizations (Figure 4) for the single-knee setup predominantly feature light colors, signifying close agreement between WBCT and MDCT models, except for minor, localized dark regions attributed to annotation variability.

#### Results of single-knee WBCT scanning

Results for the bilateral-knee WBCT scanning (Table 6) parallel those from the single-knee setup, with average absolute distances reflecting minor discrepancies across all bones. The standard deviations suggest a uniform spread of differences, reinforcing the comparability of WBCT and MDCT. Visualizations (Figure 4) continue to demonstrate predominantly light colors across the bone surfaces, confirming the overall alignment between the models in both scanning setups.

## Discussion

This study presents a detailed comparative analysis between WBCT and MDCT to ascertain the efficacy of WBCT in generating accurate 3D bone models using MDCT as a ground truth. Across both patient and cadaver specimen scans, including single-knee and bilateral-knee setups, our findings demonstrate a high consistency between WBCT and MDCT imaging modalities. This consistency is pivotal, affirming the reliability of WBCT, a relatively newer imaging technique, against the established MDCT in orthopedic diagnostics and pre-surgical planning.

A key observation from our analysis is the mix of positive and negative mean distance values across the assessed bones, indicating the absence of systemic bias of WBCT imaging compared to MDCT imaging. This balanced distribution underscores the precision and unbiased nature of both WBCT and MDCT in bone structure representation. Specifically, in the patient scans, the mean differences for the femur, tibia, fibula, and patella are remarkably small, with the patella showing mean differences as low as 0.005mm for Participant 1 and −0.01mm for Participant 4, underscoring the precision of these imaging technologies.

Notably, among all the bones, the patella showcases the smallest mean distances (e.g., −0.20mm to 0.10mm across all participants and cadavers) and standard deviation (e.g., 0.28mm to 0.55mm across all participants and cadavers). It might be due to the impact of focused region-of-interest extraction on the images of the other bones. As the patella is fully included in our scans, no FROI extraction is needed.

Visual comparisons of the 3D models further elucidate the similarities and discrepancies between WBCT and MDCT. A few local differences, marked by stark red or blue on the tips or edges of bones, point to the challenges in segmentation at these complex anatomical locations. Despite these challenges, the predominance of light coloring across most of the bone surfaces signifies minor discrepancies, affirming the overall agreement between the two imaging modalities.

While our study focuses on comparing WBCT with MDCT using traditional energy-integrating detector (EID)-based scanners, future studies should investigate how Photon-counting CT (PCCT)-based MDCT compares to WBCT in terms of spatial accuracy and diagnostic utility. PCCT represents a significant advancement in CT imaging^22,23^, offering higher spatial resolution, improved contrast-to-noise ratio, and lower radiation exposure compared to conventional EID-based MDCT. In orthopaedic imaging, PCCT can enhance the visualization of fine bone structures and potentially provide insight to the health of the bone to inform an optimal implant type and surgical technique.

Radiation dose values for all scans are summarized in Table 1 and Table 2. The lowest dose acquisitions were from the Siemens SOMATOM go.Now MDCT cadaver scans, while the highest doses were associated with the GE LightSpeed VCT. The WBCT device delivered dose levels comparable to the Siemens SOMATOM go.Now MDCT systems and considerably lower than those from the Siemens SOMATOM Force CT and GE LightSpeed VCT. Although WBCT has historically been considered a lower-dose modality, recent advances in ultra low-dose MDCT protocols, combined with iterative reconstruction techniques, have narrowed the gap for extremity imaging. Despite these variations in dose, our findings show that WBCT and MDCT produced similarly accurate 3D bone models. This suggests that the segmentation process and model accuracy are relatively robust to dose-related image noise. The ability to achieve accurate bone models across a range of dose settings supports the feasibility of using both WBCT and MDCT in low-dose clinical workflows.

However, the study is not without limitations. All scans in this study were reconstructed using bone kernels, which are designed to enhance high-frequency spatial detail. The CBCT device utilized a standard back-projection reconstruction algorithm, whereas the MDCT scanners likely employed iterative reconstruction techniques. Surgical planning software routinely accepts scans from a wide range of CT systems and reconstruction settings. This clinical flexibility, together with the consistent accuracy we observed across different scanners, suggests that 3D bone model quality is relatively robust to such variations. Nevertheless, future studies should investigate the effects of different reconstruction kernels and algorithms on quantitative bone analysis in a more controlled setting.

Although we employed a multi-stage review process to minimize segmentation error, another limitation of this study is the absence of a formal repeatability assessment for the segmentation process. Future work could incorporate intra- and inter-observer repeatability evaluations using quantitative metrics such as the Dice similarity coefficient or intraclass correlation coefficient to further assess segmentation consistency. The manual segmentation process, while ensuring high accuracy, presents a significant bottleneck for the widescale clinical adoption of 3D bone modelling. This labor-intensive step would be particularly cumbersome in settings requiring rapid turnaround, such as clinical practice or when multiple scans need to be analyzed concurrently. The pursuit of (semi-)automated segmentation techniques emerges as a necessary frontier to expedite the 3D modelling process without compromising on accuracy^24,25^. Such advancements are crucial for integrating 3D bone modelling into routine clinical practice, especially for applications demanding high precision, like pre-surgical planning. Furthermore, with the recent development of upright MDCT systems, future studies should conduct comparative analyses with upright MDCT data to evaluate the accuracy of weight-bearing cone-beam CT and provide more comprehensive validation in clinical settings.

## Conclusion

In conclusion, our study validates the comparability of WBCT and MDCT in producing 3D bone models, indicating that WBCT has the potential to provide appropriate image quality for the clinical evaluation of bone joints. While challenges in segmentation highlight areas for technological improvement, the absence of systemic bias and the high degree of consistency across imaging modalities reinforce their value in orthopedic diagnostics. Future research directed towards refining segmentation techniques and enhancing the automation of 3D modelling processes will be instrumental in unlocking the full potential of these advanced imaging technologies of weight-bearing CT.

## Data Availability

The datasets used and/or analyzed during the current study are available from the corresponding author upon reasonable request.
